# Pesticidal Activity of Sundarban Mangrove Plant Extracts against *Sitophilus* Pests and Identification of Active Constituents Using LC-MS

**DOI:** 10.1155/2021/1540336

**Published:** 2021-12-15

**Authors:** Md. Abdur Rahman, Rinku Rani Paul, Chaina Biswas, Hakima Akter, Razina Rouf, Sushmita Nath, Jamil A. Shilpi, Lutfun Nahar, Stayajit D. Sarker, Shaikh Jamal Uddin

**Affiliations:** ^1^Pharmacy Discipline, Life Science School, Khulna University, Khulna 9208, Bangladesh; ^2^Department of Pharmacy, Faculty of Life Science, Bangabandhu Sheikh Mujibur Rahman Science and Technology University, Gopalganj 8100, Bangladesh; ^3^Centre for Natural Products Discovery, School of Pharmacy and Biomolecular Sciences, Liverpool John Moores University, James Parsons Building, Byrom Street, Liverpool L3 3AF, UK; ^4^Laboratory of Growth Regulators, Institute of Experimental Botany ASCR & Palacký University, Olomouc, Czech Republic

## Abstract

Plants act as a rich source of novel natural pesticides. In the backdrop of the recent revival of interest in developing plant-based insecticides, this study was carried out to investigate the pesticidal activity of Sundarban mangrove plants. A total of nine different plant parts from five plants, namely, *Aegiceras corniculatum*, *Excoecaria agallocha*, *Heritiera fomes*, *Xylocarpus moluccensis,* and *Xylocarpus granatum*, were extracted with methanol and tested for insecticidal activity against two common stored product pests *Sitophilus oryzae* and *Sitophilus zeamais* using direct contact feeding deterrent wafer disc method. Three bark extracts from *A. corniculatum*, *E. agallocha,* and *H. fomes* showed potent and statistically significant insecticidal activity against both *S. oryzae* and *S. zeamais* pests (80–100% mortality). All the active bark extracts were further fractionated using C-18 solid-phase extraction (SPE) columns and tested for their insecticidal activity against *S. oryzae* pest to identify the active fraction. Only the SPE4 fraction (100% MeOH) from all the three active plants showed the activity against *S. oryzae* pest with a lethal concentration 50% (LC_50_) value of 0.5, 1.0, and 1.5 mg/disc for *A. corniculatum*, *E. agallocha,* and *H. fomes*, respectively. The active fraction of *A. corniculatum* was further profiled for identification of active compounds using LC-ESI-MS and identified (along with some unknown peaks) two previously reported compounds at *m/z* 625.17630 (isorhamnetin 3-*O*-rutinoside) and 422.25346 (paspaline) as major constituents. Insecticidal activities of these plants are reported in this study for the first time and would be useful in promoting research aiming for the development of new biopesticides from mangrove plants.

## 1. Introduction

The theme of World Food Day 2018 was “Zero Hunger,” and that objective was aimed to be accomplished by 2030. One of the major ways to eliminate world hunger and poverty is to make agriculture more environmentally sustainable. The pest problem is a major constraint for achieving higher production of agricultural crops [1]. About 10–30% of crops including fruits and vegetables are lost due to pests and associated diseases each year [2–5]. Management of agricultural pests over the past half century has largely depended on the use of synthetic chemical pesticides for field and postharvest protection of crops [6]. Many synthetic pesticides are banned or restricted under international agreements. Applied pesticide residues can remain in water, food, fruits, and vegetables [7]. Moreover, they also kill nontarget arthropods and insects involved in pollination. Excess use of synthetic pesticides also led to other problems including developing resistance [8]. Epidemiological studies have found carbamates and organophosphates to be carcinogenic, mutagenic, teratogenic, or allergenic [9]. Long-term residual deposition of these toxic chemicals on vegetables and fruits is likely to create different diseases in humans including cancer, skin diseases, hypertension, and kidney diseases [10]. The incidence of pesticide poisoning has been increasing, and it is estimated that about 5 million people die every year as a result of intentional, accidental, and occupational exposure worldwide [11]. Therefore, there is an urgent need to discover novel natural pesticides that could prevent damage to agricultural crops and are nontoxic to the environment.

Natural pesticides compared to synthetic ones are safer for the environment, relatively nontoxic to health, biodegradable, less expensive, easily processed, and potentially suitable for use in integrated pest management [12]. A number of plants around the world have been used traditionally for their pesticidal activity and act as a rich source of novel natural pesticides [13]. A number of examples of currently used known natural pesticides from plants are pyrethrin and pyrethroids. Neem, another most common example, Azadirachta indica, is presently the most important botanical insecticide in use throughout the world [14]. More than 2,000 different plants are known to have insecticidal properties [15]. Many other plant species have already identified to be used as natural pesticides or insecticide compounds [16–19].

Plants from mangrove and mangrove associates have long been widely used for medicinal and nonmedicinal purposes throughout the world. A literature study proved that extracts from plants of mangrove origin possess bioactivity against humans, animals, and plant pathogens [20]. The diversity in the activity of these plants could be due to the peculiar environment (high moisture, large tidal difference, high salinity, an abundance of living organisms and insects, etc.) in which they exist, producing stressful conditions, which might change their morphology, physiognomy, and biosynthetic pathways to survive [20,21]. Several novel bioactive constituents belonging to diverse chemical classes have been characterized from mangroves, and therefore, they have clinical, toxicological, and economic importance. Bangladesh has the largest single block of the Sundarban mangrove forests in the world, which is a globally significant ecosystem rich in plant biodiversity [20,22]. A total of 245 genera and 334 plant species were recorded in the Sundarban mangrove forests in 1903 [23]. Many of these Sundarban mangrove species including *Aegiceras corniculatum*, *Excoecaria agallocha*, *Heritiera fomes*, *Xylocarpus moluccensis,* and *Xylocarpus granatum* have been used in traditional medicines, but the scientific information about the biological effects of these plants and active substances are scarce and poorly documented. These plant extracts have been found to possess different bioactivity and toxicity, and a number of bioactive constituents have also been isolated from plants of mangrove origin (Supplementary Table S1). However, the chemistry, bioactivity, and efficacy of these mangrove plants and their constituents remain underexplored. For the first time, we have screened a total of nine different plant parts from *A. corniculatum*, *E. agallocha*, *H. fomes*, *X. moluccensis,* and *X. granatum* for insecticidal activity against two common stored product pests *S. oryzae* and *S. zeamais* and further identified the active constituents present in the active extract.

## 2. Materials and Methods

### 2.1. Sundarban Plants Collection, Drying, and Extraction

With the help of the Department of Forestry, Khulna, a total of nine plant parts of five (05) Sundarban mangrove plants from four families including *Aegiceras corniculatum*, *Excoecaria agallocha*, *Heritiera fomes*, *Xylocarpus moluccensis,* and *Xylocarpus granatum* have been collected from West Sundarban, Kolagachia forest range, Munshiganj, Satkhira (22.2152°N, 89.2376°E), Bangladesh (Table 1 and Supplementary Figure S1). All the collected plants were identified by Prof. Dr. Md. Azharul Islam, Forestry and Wood Technology Discipline, Khulna University, and immediately air-dried for further extraction. The dried plant parts were grinded into a coarse powder with the help of a suitable grinder. The powders were stored in airtight containers and kept in the cool, dark, and dry place until analysis commenced. About 175 g of each powdered material was taken in clean, flat-bottomed glass containers, and the material was soaked with 900 ml methanol. The containers with their contents were sealed and kept for a period of 7 days accompanying occasional shaking and stirring. The whole mixture then underwent a coarse filtration by a piece of clean, white cotton. Then, it was filtered through Whatman filter paper. Following the filtration, the solvent was evaporated using rotary evaporation.

### 2.2. Chemical Reagents and Equipment

All the solvents used for extraction and chromatographic use were analytical grade and HPLC grade from Merck KGaA, Germany. The positive control permethrin was bought from a local supermarket. The solid-phase fractionation was conducted with reverse-phase SPE columns (Alltech, 60 mL, 10 g high capacity C_18_) using Visiprep 12-port vacuum manifold (Sigma-Aldrich, Germany). Chemical profiling of active fraction was analyzed with an Agilent 1260 Infinity II series HPLC system (Agilent Technologies Inc., Santa Clara, CA, USA) combined with Agilent 6530 Accurate-Mass Q-TOF-LC-MS mass spectrometer (Agilent Technologies Inc., Santa Clara, CA, USA). The HPLC system had a binary solvent manager, a column, and a photodiode array detector. The column was a 50 mm × 2.1 mm i.d., 1.8 *μ*m, Agilent ZORBAX Eclipse Plus C_18_ reverse-phase column (Agilent Technologies Inc., Santa Clara, CA, USA).

### 2.3. Solid-Phase Fractionation of Active Plant Extracts

Solid-phase extraction (SPE) is widely employed as an alternative method to liquid-liquid extraction aiming at the separation, purification, and concentration of bioactive compounds. The important application of SPE is the fractionation of the crude extract into different compounds or groups of compounds by eluting the extract with different solvents for further chromatographic separations [24]. The crude active methanolic extracts from *A. corniculatum*, *E. agallocha*, and *H. fomes* were fractionated into four fractions using reverse-phase SPE columns (Alltech, 60 mL, 10 g high capacity C18) with a stepwise methanol/water gradient. SPE columns were first preconditioned with 3 bed volumes (BV) of methanol (180 mL) and then equilibrated using 3 BV of water (180 mL). A sample of each crude methanolic extract (500 mg) was first dissolved in 1 mL of methanol and loaded into the SPE column and then fractionated into four fractions by eluting with water/methanol using a stepwise gradient (Supplementary Table S2).

### 2.4. Insect Tested

Insecticidal activity of collected mangrove plant extracts was tested against stored product insects *Sitophilus oryzae* and *Sitophilus zeamais*(family: Dryophthoridae). The pests were collected from the Agrotechnology Discipline, Khulna University, and the culture was maintained in the laboratory on rice grain and red lentil grain, respectively, in plastic containers (26 cm × 30 cm × 20 cm) at 29 ± 1 °C, 50–60% r.h., and a 16 : 8 light:dark cycle photoperiod without exposure to any insecticide.

### 2.5. Pesticidal Activity Screening of the Sundarban Plant Extracts

The insecticidal activity of the Sundarban plant extracts was investigated against two common stored product pests (*Sitophilus oryzae* and *Sitophilus zeamais*) as per the methods of Boussaada et al., 2008 and Kim et al., 2003 with some modification [12,25]. Briefly, a dose of 2.5 mg of each plant extract in 400 *μ*l MeOH was applied to wafer discs made of wheat (weight about 30 mg and 1 cm diameter). Permethrin at 2.0 mg/discs was used as a positive control, and only vehicle (400 *μ*l MeOH/discs) served as a negative control in the experiment. The discs were left under a fume hood to dry, and then, each disc was weighed before placement in the Petri dish for activity study. After drying, discs were placed in Petri dish (9 cm diameter) and then 10 adults of each either *S. oryzae* or *S. zeamais* were placed in the Petri dish, which was covered with a lid and kept at 29 ± 1 °C, 50–60% r.h., and a 16 : 8 light:dark cycle. Mortalities were determined by the number of dead adults at 5, 10, and 15 days after treatment. Test insects were considered dead if appendages did not move when prodded with a fine brush. Three replications were setup for ach assay.

### 2.6. LC_50_ Determination of Active Fraction

All the fractions were further evaluated for pesticidal activity using the above method against *S. oryzae* pest, and lethal concentration 50% (LC_50_) of all active fractions was determined by testing each fraction at a dose of 2.5, 2.0, 1.5, 1.0, and 0.5 mg/disc. LC_50_ was recorded at which concentration 50% of the pest was died.

### 2.7. LC-ESI-MS Analysis of *A. corniculatum* Active Fraction

Chemical profiling of active SPE4 fraction of *A. corniculatum* was analyzed with an Agilent 1260 Infinity II series HPLC system (Agilent Technologies Inc., Santa Clara, CA, USA) combined with Agilent 6530 Accurate-Mass Q-TOF-LC-MS mass spectrometer (Agilent Technologies Inc., Santa Clara, CA, USA). The HPLC system had a binary solvent manager, a column, and a photodiode array detector. The column was a 50 mm × 2.1 mm i.d., 1.8 *μ*m, Agilent ZORBAX Eclipse Plus C_18_ reverse-phase column (Agilent Technologies Inc., Santa Clara, CA, USA). Elution was carried out with a binary solvent system consisting of 0.1% aqueous formic acid (*A*) and methanol containing 0.1% formic acid (B) at a constant flow rate of 0.4  *μ*L/min. The elusion events were as follows: 0 min: 5% B; 0–7.0 min: 5–100% B; 7.0–10.0 min: 100% B; 10.0–11.0 min: 100–5% B; and 11.0–13.0 min: 5% B. UV-Vis (190–500 nm), and MS data (*m*/*z* 100 to 1700) were recorded from 0 to 11 min. The ESI ionization was performed in positive ion mode, and the instrument parameters were as follows: capillary voltage: 2.4 kV; desolvation temperature: 650°C; source temperature: 150°C; desolvation and cone gas (N_2_) flow rate: 1,000 and 100 Lh^−1^, respectively; and collision gas: argon.

## 3. Results

### 3.1. Insecticidal Activity of Collected Sundarban Plant Crude Methanolic extracts

Plant-derived insecticides are commonly pest-specific and are relatively nontoxic to nontarget organisms including humans [26]. A number of mangroves and mangrove associates are used as folklore medicinal, insecticidal, and pesticidal plants [27]. In this study, a total of nine extracts from five Sundarban mangrove plants were investigated for insecticidal activity against two store product pests *S. oryzae* and *S. zeamais* that so far not studied before. For the screening of insecticidal activity, all the plant parts were extracted with methanol and Table 1 represents the extraction yields of all collected plants. Extraction yield results demonstrated that *X. granatum* and *X. moluccensis* showed the highest yield indicating a high number of polar constituents in comparison to *A. corniculatum, H. fomes,* and *E. agallocha* (Table 1).

Insecticidal activities of crude extracts were tested against two store product pests *S. oryzae* and *S. zeamais.*Table 2 demonstrates the toxicity of the methanolic crude extracts against the tested store product pests at 2.5 mg/disc for 5, 10, and 15 days of treatment. The results demonstrated that the nonselective mortalities were higher in bark extracts of *A. corniculatum, E. agallocha,* and *H. fomes* (80–100% mortality) against both *S. oryzae* and *S. zeamais* pests throughout the treatment period at a dose of 2.5 mg/disc (Table 2). The methanol extracts of bark and leaf of *X. moluccensis* showed selective insecticidal activity against *S. oryzae* (50% mortality) and *S. zeamais* (73% mortality), respectively, at 15 days of treatment. Other extracts showed low to no insecticidal activity against either of the tested pests. The bark extracts of *A. corniculatum, E. agallocha,* and *H. fomes* showed over 42% mortality after the 5 days of treatment, the value increased to over 82–100% mortality after 15 days of treatment against both the pests, and the data were statistically significant (*P* < 0.001), whereas the standard permethrin showed 80% to 100% mortality at 5th to 15th days of treatment.

### 3.2. SPE Fractionation of Active Crude Extracts and Insecticidal Activity of Each Fraction

Primary pesticidal activity results demonstrated that the crude extract of *A. corniculatum, E. agallocha,* and *H. fomes* possesses significant pesticidal activity against the tested pests. Solid-phase extraction (SPE) is a widely employed fractionation technique aiming at the separation, purification, and concentration of bioactive polar compounds for further chromatographic separations [24]. The active crude methanolic extracts were further fractionated into four fractions (SPE1, SPE2, SPE3, and SPE4) using stepwise gradient methanol/water using C18 cartridge and further tested for pesticidal activity against *S. oryzae*. The results demonstrated that only the SPE4 fraction (100% MeOH) for all the tested plant extracts was active against *S. oryzae* at 2.0 mg/disc.  Table 3 presents the toxicity results of SPE4 fractions of *A. corniculatum, E. agallocha,* and *H. fomes* against *S. oryzae* pest. The percentage of mortality of the SPE4 fraction for *A. corniculatum* was found the highest (97%) at 2.0 mg/disc among the fractions tested. The other two SPE4 fractions of *E. agallocha* and *H. fomes* also showed 90% and 80% mortality, respectively, after 15 days treatment period (Table 3). The insecticidal effect of *A. corniculatum* fractions was also prominent after the initial 5 days treatment (37% mortality), and that value was increased to 73% at 10 days and 97% at 15 days treatment. All the results were statistically significant. Further LC_50_ concentration was determined for all of the active SPE4 fractions, and *A. corniculatum* fraction was found the lowest LC_50_ (0.5 mg/disc) among all the fractions tested (LC_50_: 1–1.5 mg/disc) and was comparable to permethrin (LC_50_ : 0.4 mg/disc) (Table 3). This result suggests that the potent pesticide constituents present in the SPE4 fraction of *A. corniculatum*.

### 3.3. LC-ESI-MS Identification of Active Compounds in *A. corniculatum* Active Fraction

**Figure 1:**
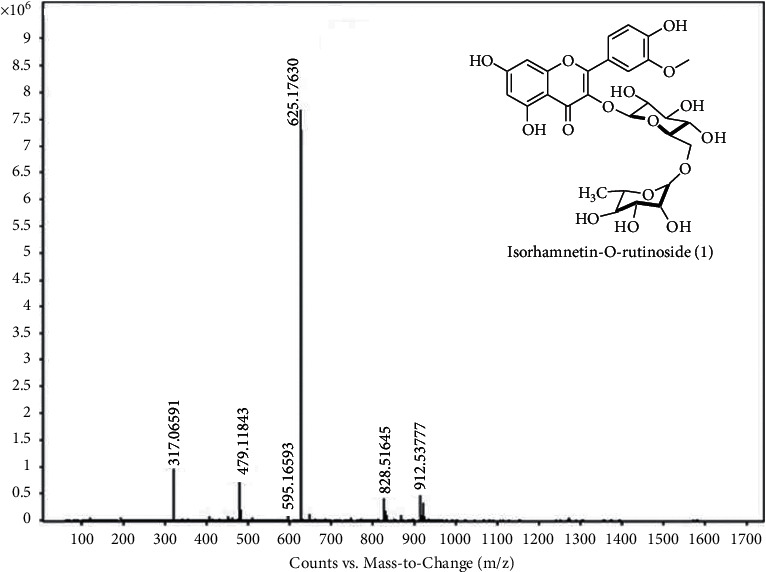
LC-ESI-MS chromatogram of a base peak [*M*+ *H*]^+^ with *m*/*z* 625.17598 at 6.05 min present in SPE4 fraction of *A. corniculatum* for the identified flavonol compound (**1**).

**Figure 2:**
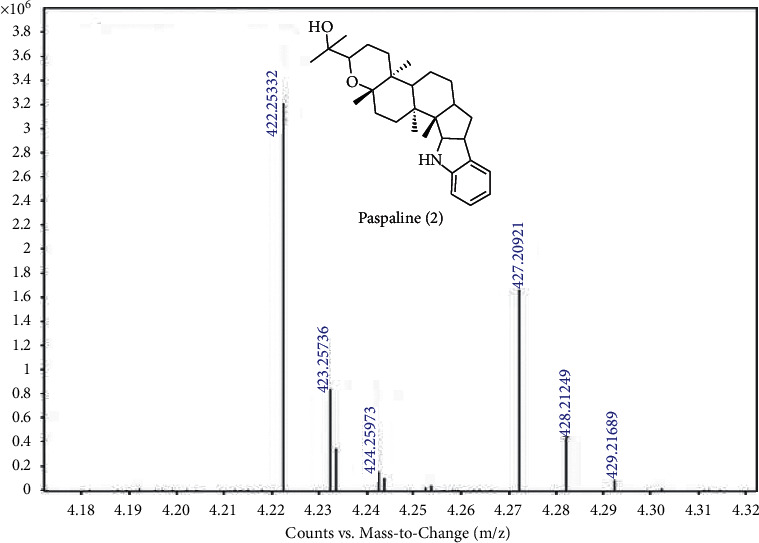
LC-ESI-MS chromatogram of a base peak [*M*+ *H*]^+^ with *m/z* 422.25332 at 7.84 min present in SPE4 fraction of *A. corniculatum* for the identified indole-diterpenoid compound (**2**).

Plants have the ability to develop different constitutive and inducible mechanisms for the protection from pathogen and herbivores via various secondary chemical compounds or morphological barriers [28]. A number of plant secondary metabolites have also been reported to use for the management of pests [29]. Liquid chromatography coupled to mass spectrometry (LC-MS) technique has become more sensitive and specific for the analysis of phytoconstituents. In this study, the most active SPE4 fractions of *A. corniculatum* were further analyzed by LC-MS using electrospray ionization (ESI) positive ion mode. Chemical constituents reported so far from these plants were matched with the molecular ion [*M*+*H*]^+^ peaks observed in the mass spectrums (Figures 1 and 2) of SPE4 fraction of *A. corniculatum,* and the results showed that the active SPE4 fractions of *A. corniculatum* contain different classes of phytoconstituents.

The LC-ESI-MS mass chromatogram of the active SPE4 fraction of *A. corniculatum* revealed two major peaks. Compound **1** in the SPE4 fraction of *A. corniculatum* exhibited a base peak [*M*+ *H*]^+^ with *m*/*z* 625.17598 at 6.05 min that suggested a molecular formula [C_28_H_32_O_16_], which confirmed that compound **1** was isorhamnetin 3-*O-*rutinoside (flavonol) m.w. 624.16870 previously identified from bark extract of *A. corniculatum* [30] (Figure 1). The identification of compound **1** was further confirmed by comparing its mass fragmentation of pseudomolecular ion (*m/z* at 625 [*M*+*H*]^+^) of isorhamnetin 3-*O*-rutinoside that resulted in major fragments including a complete loss of disaccharide, rutinose at *m/z* 317 ([(*M* + *H*)-308]^+^), from flavonol rutinosides, which has been previously reported [31] (Figure 1), whereas compound **2** in *A. corniculatum* fraction appeared as a base peak [*M*+ *H*]^+^ with *m/z*422.25332 at 7.84 min that suggested a molecular formula [C_28_H_39_NO_2_] and confirmed compound **2** as paspaline (indole-diterpenoid) m.w. 421.2460, previously identified from bark extract of *A. corniculatum* [32] (Figure 2). Paspaline was purified from the endophytic fungus *Penicillium* sp. that isolated from the mangrove plant *Aegiceras corniculatum* [33].

## 4. Discussion

Mangrove plants grow under hostile stress conditions, and their distinctive adaptability features make them produce diverse bioactive secondary constituents that play an essential role in plant defense and survival [34]. With the aim of exploring the potential of mangrove botanical insecticides against store product pests, this is the first time we screened five Sundarban plant extracts for their toxic effect against two store product pests *S. oryzae* and *S. zeamais.* These store product insects are widely distributed pests that cause heavy damage by infesting grain. These insects were broadly used in the study of botanical extracts as insect repellents [35,36]. Insecticidal screening of all crude plant extracts at 2.5 mg/disc against the pests revealed *A. corniculatum, E. agallocha,* and *H. fomes* extracts were the most susceptible to both the tested pests as they showed the highest mortality. Compared to controls, an 80–100% mortality was observed by the above three crude extracts and selected further for fractionation and bioassays (2.0 mg/disc). The solid-phase fractionation was performed to concentrate the active compounds present in the crude extracts. All the SPE fractions were tested for their toxicity against *S. oryzae* pest, and the highest bioactivity (80–97% mortality) against the pest was mainly observed by the SPE4 (100% MeOH) fraction for all the plant extracts. Compared to the SPE4 fractions of all these three plant extracts, the SPE4 fraction of *A. corninulatum* was more toxic to the *S. oryzae* with the lowest LC_50_ values (0.5 mg/disc) than other tested fractions (LC_50_: 1–1.5 mg/disc). The mangrove plant species that are distributed around the world have economic and medicinal importance [34]. Traditional reports stated that extracts and chemicals from mangroves were commonly used as bush medicine for a long time and insecticide and piscicide activities [27]. A number of studies available on the activity of mangrove botanical extracts from *A. corniculatum* and *E. agallocha* and *H. fomes* possess antioxidant, antimicrobial, antiulcer, cytotoxic, and antitumor activities [37–40]. There was no previous insecticidal report on these plant extracts, but their bactericidal and cytotoxic activity might relate to their insecticidal activity. Plants species reported to use as fish poison could have insecticidal activity [41]. Several mangrove species have been used as fish poison [27], and among these plants, the extract from different parts of *E. agallocha* reported fish poison activity, which might support its insecticidal activity [37]. Furthermore, constituents with pesticidal properties have also been reported from fresh twigs and barks of *A. corniculatum*, *E. agallocha,* and *Heritiera* sp. that showed metabolic toxicity against different fish [27].

A number of bioactive constituents, such as polyphenols, flavonoids, triterpenes, essential oils, limonoids, coumarins, terpenoids, glycosides, and alkaloids, have been isolated from plants of mangrove origin [27,34]. It is practically difficult to specifically relate the sensitivity of the crude extract against insects as these botanical extracts generally represent a complex mixture of compounds. Some of these phytoconstituents are known to possess insecticidal activity, which could explain the observed toxicity of these plant extracts. The previous report stated that an atisane-type diterpene, ent-16*α*-hydroxy-atisane-3,4-lactone, was isolated from the bark of *E. agallocha* that exhibited significant antifouling activity against the adherence of *Pseudomonas pseudoalcaligenes* [42]. On the other hand, *E. agallocha* reported to possess phorbol ester [43], a potent insect repellent [44]. These reported constituents might be responsible for *E. agallocha's* insecticidal activity. *H. fomes* has different traditional uses other than insecticides although the plant reported to contain different polyphenolics including procyanidins [45]. Interestingly, several reports stated that polyphenol-rich extract possesses insecticidal activity [46] and this could be the case for the potent insecticidal activity of *H. fomes*.

The results of this study revealed that *A. corniculatum* bark extracts could serve as a source of potential insecticidal lead molecules as they showed the most significant insecticidal activities against both the tested pests. Therefore, the active SPE4 fraction of *A. corniculatum* was further analyzed by LC-MS to identify active principles responsible for its insecticidal activity. Two compounds, namely, isorhamnetin 3-*O*-rutinoside (**1**) and paspaline (**2**), were identified as main constituents in the active SPE4 fraction along with some unknown peaks. Compounds **1** and **2** are flavonol and indole-diterpenoid, respectively, which were previously isolated from the bark of *A. corniculatum* (Figures 1 and 2) [30,32]. Flavonols are among the most abundant natural phenolics that have strong antioxidant properties and thought to play a role in protecting plants from microorganisms, insects, and larger herbivores [47]. There was no single study on compound **1** for its insecticidal activity, but a number of studies showed that extracts containing isorhamnetin-derived flavonoids possess insecticidal activity. For example, the flower of Calendula officinalis is rich in isorhamnetin 3-O-rutinoside flavonoid and showed insecticidal activity against milk weed bugs [48]. Other reports demonstrated that *O*-methyl flavonoids including isorhamnetin from Calceolaria integrifolia were the most active secondary metabolites that act as biopesticides [49]. The other identified compound paspaline (2) was an indole-diterpenoid mycotoxin and was not studied before for its insecticidal activity against store product pests. However, previous toxicity investigation of compound 2 against the hemiptera (*Oncopeltus fasciatus*) and dipteran (*Ceratitis capitata*) did not find any toxicity against these pests [50]. Furthermore, *A. corniculatum* is reported to possess benzofuran derivatives [51], which have been reported to possess insecticidal activity [52]. However, we could not identify any benzofuran derivative in the active SPE4 fraction of *A. corniculatum,* rather a benzopyrano-indole derivative paspaline (**2**) was identified. Therefore, the insecticidal activity of *A. corniculatum* might be due to its polyphenolic constituents and could be a new source of novel biopesticides.

## 5. Conclusions

The bark extracts of Sundarban plants *A. corniculatum*, *E. agallocha,* and *H. fomes* are known to exhibit a number of therapeutic activities and are already known to have considerable medicinal importance. This study demonstrated that the botanical extracts from all these three plants showed high toxicity against the tested store product pests, especially the bark extract of *A. corniculatum* with low LC_50_ against *S. oryzae*. The active fraction of *A. corniculatum* was further studied for identification of the active compounds using LC-ESI-MS analysis and identified two known compounds isorhamnetin 3-*O*-rutinoside and paspaline as major constituents. Although these two compounds are pretty common in some medicinal plants, there is no report to date on their insecticidal activity. Therefore, the insecticidal activity of *A. corniculatum* might be due to its known constituents and could be a new source of novel biopesticides. The results of this study would be useful in promoting research aiming for the development of new biopesticides from mangrove plants, especially *A. corniculatum* against store product pests. Furthermore, molecular mechanistic study needs to be performed on these bioactive compounds for the development of effective natural pesticides.

## Figures and Tables

**Table 1:** List of collected Sundarban plant species for methanol extraction, their traditional uses, part used, and extraction yields [24]].

Plant name	Family	Local name	Traditional uses	Part used	Extraction yield (%)^∗^
*Aegiceras corniculatum* (L.) blanco	Myrsinaceae	Kholisha	Rheumatism, painful arthritis, inflammation, and fish poison	Bark	13.5
*Excoecaria agallocha* (L.)	Euphorbiaceae	Geoa	Epilepsy, conjunctivitis, dermatitis, hematuria, leprosy toothache, and fish poison	Bark	7.2
*Heritiera fomes* (buch.)	Sterculiaceae	Sundari	GIT disorders, hepatic disorders, skin diseases, diabetes, and goiter	Bark	8.9
				Leaf	10.2
*Xylocarpus granatum* (K. D. Koenig)	Meliaceae	Dhundul	Antibacterial, malaria, inflammation, dysentery, diarrhea, cholera, CNS depressant, and anticancer	Bark	17.1
*Xylocarpus moluccensis* (lam.) M. Roem.	Meliaceae	Pashur	Fever, inflammation, dysentery, diarrhea, cholera, and abdominal problems	Bark	14.1
				Leaf	4.7
				Fruit	13.3
				Pneumatophore	23.2

^*∗*^ Extraction yield (%) = dry weight of methanol extract/dry weight of test plant part) x100.

**Table 2:** Insecticidal activity of the methanolic crude extract of Sundarban plants against *S. oryzae* and *S. zeamais* insects at 2.5 mg/disc concentration.

Name of plant extract	Plant part^¥^	No. of insect	No of dead adult *S. oryzae* insects ^*∗*^			No of dead adult *S. zeamais* insects ^*∗*^		
			Days after treatment			Days after treatment		
			5^th^	5^th^	15^th^	5^th^	5^th^	15^th^
*A. corniculatum* (L.) blanco	B	10	6.0 ± 0.6^*∗*^	8.3 ± 0.3^*∗*^	9.6 ± 0.3^*∗*^	5.0 ± 0.5^*∗*^	6.0 ± 0.6^*∗*^	8.3 ± 0.8^*∗*^
*E. agallocha* (L.)	B	10	7.7 ± 0.3^*∗*^	9.0 ± 0.6^*∗*^	10 ± 0.0^*∗*^	4.3 ± 0.3^*∗*^	8.0 ± 0.5^*∗*^	9.3 ± 0.6^*∗*^
*H. fomes* (buch.)	B	10	4.6 ± 0.3^*∗*^	8.3 ± 1.6^*∗*^	10 ± 0.0^*∗*^	5.0 ± 0.6^*∗*^	6.3 ± 0.8^*∗*^	10 ± 0.0^*∗*^
	L	10	1.3 ± 0.3	2.3 ± 0.3	3.6 ± 0.8^∗^	0.0 ± 0.0	1.3 ± 0.33	3.6 ± 0.3
*X. granatum* (K. D. Koenig)	B	10	1.0 ± 0.0	1.3 ± 0.3	2.0 ± 0.5	0.0 ± 0.0	0.33 ± 0.3	1.3 ± 0.33
*X. moluccensis* (lam.) M. Roem.	B	10	2.3 ± 0.3	3.0 ± 0.5	5.0 ± 0.6^*∗*^	0.33 ± 0.3	1.0 ± 0.0	1.6 ± 0.3
	L	10	0.3 ± 0.3	1.0 ± 0.6	1.6 ± 0.3	1 ± 0.6	2.6 ± 0.33	7.3 ± 1.4^*∗*^
	F	10	0.6 ± 0.3	1 ± 0.0	2.3 ± 0.3	0.0 ± 0.0	0.0 ± 0.0	1.3 ± 0.3
	P	10	0.0 ± 0.0	1 ± 0.6	2.3 ± 1.4	0.0 ± 0.0	1.3 ± 0.3	2.0 ± 0.5
Permethrin (2.0 mg/disc)	-	10	7.6 ± 0.3^*∗*^	8.3 ± 0.4^*∗*^	10 ± 0.0^*∗*^	8.0 ± 0.6^*∗*^	9.0 ± 0.5^*∗*^	10 ± 0.0^*∗*^
Control disc (vehicle)	-	10	0.0 ± 0.0	1.3 ± 0.3	1.3 ± 0.3	0.0 ± 0.0	1.3 ± 0.3	2.0 ± 0.6

¥ *B*= Bark, *L* = leaf, *F* = fruit, *P* = pneumatophore; ^*∗*^ results are expressed as mean ± SEM. ^*∗*^*p* < 0.001 vs. control.

**Table 3:** Insecticidal activity of the SPE4 fractions of *A. corniculatum, E. agallocha,* and *H. fomes* against *S. oryzae* insect at 2.0 mg/disc and their LC_50_.

Name of plant	Fraction	No. of insects	No of dead adult insects ^*∗*^			% of mortality	Lethal concentration 50% (LC_50_) mg/disc
			Days after treatment				
			5^th^	10^th^	15^th^		
*A. corniculatum* (L.) blanco	SPE4	10	3.7 ± 0.3^*∗*^	7.3 ± 0.47	9.7 ± 0.5^*∗*^	97	0.5
*E. agallocha* (L.)	SPE4	10	2.0 ± 0.6^*∗*^	5.3 ± 0.5^*∗*^	9.0 ± 0.7^*∗*^	90	1.0
*H. fomes* (buch.)	SPE4	10	2.7 ± 0.3^*∗*^	4.3 ± 0.3^*∗*^	8.0 ± 0.6^*∗*^	80	1.5
Pemethrin	-	10	5.6 ± 0.8^*∗*^	6.3 ± 1.2^*∗*^	10 ± 0.0^*∗*^	100	0.4
Control (vehicle only)	-	10	0.0 ± 0.0	0.7 ± 0.3	1.0 ± 0.5	10	-

^*∗*^ Results are expressed as mean ± SEM. ^*∗*^*p* < 0.001 vs. control.

## Data Availability

All relevant data within this manuscript are fully available without any restriction.

## Supplementary Materials

Supplementary Materials Table S1: list of collected Sundarban mangrove plants with their traditional use, reported activity, and isolated constituents. Table S2: water/methanol gradient for SPE fractionation of active methanolic crude extracts. Figure S1: GPS location of Sundarban mangrove plant collection (Kolagachia forest range, Munshiganj, Satkhira, Bangladesh) (22.2152°N, 89.2376°E). Figure S2: LC-MS chromatogram of the active SPE4 subfraction of *A. corniculatum*.
