# Supplementary material for: Pesticidal Activity of Sundarban Mangrove Plant Extracts against Sitophilus Pests and Identification of Active Constituents Using LC-MS
Source: Adv Pharmacol Pharm Sci. 2021 Dec 15;2021:1540336. doi: 10.1155/2021/1540336 (PMC8695028; doi:10.1155/2021/1540336)
Supplement: Supplementary Materials — Table S1: list of collected Sundarban mangrove plants with their traditional use, reported activity, and isolated constituents. Table S2: water/methanol gradient for SPE fractionation of active methanolic crude extracts. Figure S1: GPS location of Sundarban mangrove plant collection (Kolagachia forest range, Munshiganj, Satkhira, Bangladesh) (22.2152°N, 89.2376°E). Figure S2: LC-MS chromatogram of the active SPE4 subfraction of A. corniculatum. [file 1540336.f1.docx]

**Supplementary Materials**

**Pesticidal activity of Sundarban mangrove plant extracts against *Sitophilus* pests and identification of active constituents using LC-MS**

Md. Abdur Rahman^1,¥^, Rinku Rani Paul^1, ¥^, Chaina Biswas^1, ¥,^, Hakima Akter^1, ¥^, Razina Rouf^2^, Sushmita Nath^3^, Jamil A. Shilpi^1^, Lutfun Nahar^4^, Stayajit D. Sarker^3^, Shaikh Jamal Uddin^1*^

*^1^Pharmacy Discipline, Life Science School, Khulna University, Khulna 9208, Bangladesh*

*^2^Department of Pharmacy, Faculty of Life Science, Bangabandhu Sheikh Mujibur Rahman Science and Technology University, Gopalganj 8100, Bangladesh*

*^3^Centre for Natural Products Discovery, School of Pharmacy and Biomolecular Sciences, Liverpool John Moores University, James Parsons Building, Byrom Street, Liverpool L3 3AF, UK*

*^4^Laboratory of Growth Regulators, Institute of Experimental Botany ASCR & Palacký University, Olomouc, Czech Republic*

^¥^Authors equally contributed

*Correspondence

**Prof. Shaikh Jamal Uddin**

Pharmacy Discipline, Khulna University, Bangladesh

E-mail: uddinsj@yahoo.com

Telephone: +8801711337375

Fax: +880-41-731244

ORCID ID: 0000-0003-3163-2118

**Table S1:** List of collected Sundarbans mangrove plants with their traditional use, reported activity and isolated constituents

| **Scientific name**  **(Family)** | **Local name** | **Traditional uses** | **Chemical compounds** | **Reported Pharmacological Activity** | **References** |
| --- | --- | --- | --- | --- | --- |
| *Aegiceras corniculatum*  *(F: Myrsinaceae)* | Kholisha | Rheumatism  Painful arthritis Inflammation  Fish poison | 2-Methoxy-3-nonylresorcinol, 5-O-ethylembelin, isorhamnetin-3-O-rutinoside, 2-O-acetyl-5-O-methylembelin, 3,7-dihydroxy-2,5-diundecylnaph thoquinone, 2,7-dihydroxy-8-methoxy-3,6-diundecyl dibenzofuran-1,4-dione, 2,8-dihydroxy-7-methoxy-3,9-diundecyldibenzofuran-1,4-dione, and 10-hydroxy-4-O-methyl-2,11-diundecylgom philactone, paspaline | Antioxidant  Anti-inflammatory  Hepatoprotective  Antiplasmodial  Antiproliferative Antidiabetic Antinociceptive  Antiviral  Cytotoxic | [1-9] |
| *Excoecaria agallocha*  *(F: Euphorbiaceae)* | Geowa | Epilepsy  Conjunctivitis  Dermatitis  Hematuria  Leprosy toothache  Fish poison | Pentacyclictriterpenoid, phorbol Ester, polyphenols, dichloromethane, lignin, pentosan, α-cellulose | Antifoluing  Anti-tumor  Anti-ulcer  Antimicrobial Antioxidant  Antifungal  Antioxidant  Antifilarial | [9-14] |
| *Heritiera Fomes*  *(F: Sterculiaceae)* | Sundari | GIT disorders,  Hepatic disorders  Skin diseases  Diabetes and Goiter | Procyanidins. trimeric, pentameric, hexameric procyanidins | Antihyperglycemic  Antinociceptive  Antioxidant Antimicrobial  Anticancer  Antibacterial | [15-18] |
| *Xylocarpus moluccensis*  *(F: Meliaceae)* | Pashur | Fever  Inflammation  Dysentery  Diarrhea  Cholera  Abdominal problems | Mexicanolides, phragmalin,  Moluccensins H, xylocarpanoids A, B,6-hydroxymexicanolide, Khayanolides, limonoids, Godavarins A–C, Acetoxydihydronomili, xyloccensin O,P and Q | Antidiarrhoeal  CNS depressant  Antiviral  Antibacterial  Cytotoxic  Antioxidant | [18-22] |
| *Xylocarpus granatum*  *(F: Meliaceae)* | Dhundul | Antibacterial  Malaria,  Inflammation  Dysentery  Diarrhea  Cholera  CNS depressant  Anticancer | Akaloids viz. N-methyl flindersine, chelerythrine, dihydrochelerythrine, acetonyl dihydrochelerythrine, Limonoids: i.e., xyloccensin O, xyloccensin P, xyloccensin Q angedunin, 1α-hydroxy-1,2-dihydrogedunin, Flavonoids: catechin and epicatechin and procyanidins of the B1 | Antimicrobial  Antidiarrheal  Antioxidant  Cytotoxic | [10, 23-37] |


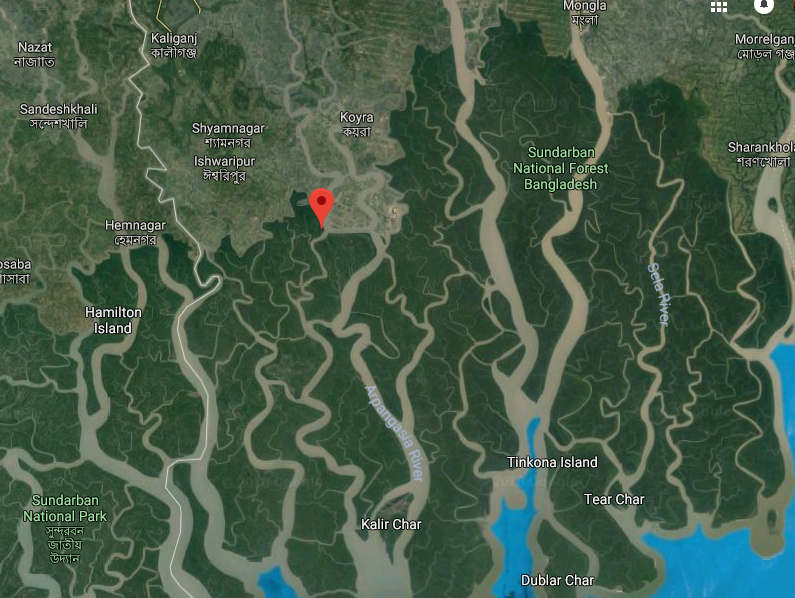


**Figure S1**: GPS Location of Sundarban mangrove plant collection (Kolagachia forest range, Munshiganj, Satkhira, Bangladesh) (22.2152°N, 89.2376°E).

**Table S2:** Water/Methanol gradient for SPE fractionation of active methanolic crude extracts.

| **Fraction** | **Solvent** | **Volume (mL)** |
| --- | --- | --- |
| SPE 1 | 100% Water | 180 |
| SPE 2 | 20% MeOH/Water | 180 |
| SPE 3 | 40% MeOH/Water | 180 |
| SPE 4 | 100% MeOH | 180 |

**Figure S2.** LC-MS chromatogram of the active SPE4 sub-fraction of *A. corniculatum*

**References:**

1. Roome, T., et al., *A study on antioxidant, free radical scavenging, anti-inflammatory and hepatoprotective actions of Aegiceras corniculatum (stem) extracts.* Journal of Ethnopharmacology, 2008. **118**(3): p. 514-521.

2. Ravikumar, S., et al., *Antiplasmodial activity of two marine polyherbal preparations from Chaetomorpha antennina and Aegiceras corniculatum against Plasmodium falciparum.* Parasitol Res, 2011. **108**(1): p. 107-13.

3. Gurudeeban, S., et al., *Antidiabetic effect of a black mangrove species Aegiceras corniculatum in alloxan-induced diabetic rats.* Journal of Advanced Pharmaceutical Technology & Research, 2012. **3**(1): p. 52-56.

4. Roome, T., et al., *Evaluation of antinociceptive effect of Aegiceras corniculatum stems extracts and its possible mechanism of action in rodents.* J Ethnopharmacol, 2011. **135**(2): p. 351-8.

5. Zhang, G., et al., *Antiviral isoindolone derivatives from an endophytic fungus Emericella sp. associated with Aegiceras corniculatum.* Phytochemistry, 2011. **72**(11-12): p. 1436-42.

6. Ding, L., H.-M. Dahse, and C. Hertweck, *Cytotoxic Alkaloids from Fusarium incarnatum Associated with the Mangrove Tree Aegiceras corniculatum.* Journal of Natural Products, 2012. **75**(4): p. 617-621.

7. Zhang, D., et al., *Oleanane triterpenes from Aegiceras corniculatum.* Fitoterapia, 2005. **76**(1): p. 131-133.

8. Rajeswari, K. and B. Tadiboina, *Aegiceras corniculatum linn (Myrsinaceae).* Journal of Chemical and Pharmaceutical Research, 2015. **7**: p. 305-316.

9. Bandaranayake, W.M., *Traditional and medicinal uses of mangroves.* Mangroves and Salt Marshes, 1998. **2**(3): p. 133-148.

10. Simlai, A. and A. Roy, *Biological activities and chemical constituents of some mangrove species from Sundarban estuary: An overview.* Pharmacognosy Reviews, 2013. **7**(14): p. 170-178.

11. Konoshima, T., et al., *Anti-tumor-promoting activity of the diterpene from Excoecaria agallocha. II.* Biol Pharm Bull, 2001. **24**(12): p. 1440-2.

12. Thirunavukkarasu, P., L. Ramkumar, and T. Ramanathan, *Anti-ulcer Activity of Excoecariaagallochabark on NSAID-induced Gastric Ulcer in Albino Rats.* Global journal of pharmacology, 2009. **3**(3): p. 123-126.

13. Patra, J.K., et al., *Screening of antioxidant and antifilarial activity of leaf extracts of Excoecaria agallocha L.* International Journal of Integrative Biology, 2009. **7**(1): p. 9-15.

14. Wang, Z.C., et al., *A new atisane-type diterpene from the bark of the mangrove plant Excoecaria agallocha.* Molecules, 2009. **14**(1): p. 414-22.

15. Ali, M., et al., *An evaluation of antihyperglycemic and antinociceptive effects of methanol extract of Heritiera fomes Buch-Ham. (Sterculiaceae) barks in Swiss albino mice.* Advances in Natural and Applied Sciences, 2011. **5**(2): p. 116-21.

16. Wangensteen, H., et al., *Antioxidant and antimicrobial effects of the mangrove tree Heritiera fomes.* Nat Prod Commun, 2009. **4**(3): p. 371-6.

17. Patra, J.K. and H. Thatoi, *Anticancer activity and chromatography characterization of methanol extract of Heritiera fomes Buch. Ham., a mangrove plant from Bhitarkanika, India.* Oriental Pharmacy and Experimental Medicine, 2013. **13**(2): p. 133-142.

18. Mondal, S., et al., *A comparative study on the in vitro antibacterial activity of the pneumatophores of Heritiera fomes and Xylocarpus moluccensis.* Ars Pharmaceutica, 2008. **49**(51-6).

19. Uddin, S.J., et al., *Antidiarrhoeal activity of the methanol extract of the barks of Xylocarpus moluccensis in castor oil- and magnesium sulphate-induced diarrhoea models in mice.* J Ethnopharmacol, 2005. **101**(1-3): p. 139-43.

20. Sarker, S.D., et al., *Neuropharmacological properties of Xylocarpus moluccensis.* Fitoterapia, 2007. **78**(2): p. 107-111.

21. Pudhom, K., et al., *Moluccensins H−J, 30-Ketophragmalin Limonoids from Xylocarpus moluccensis.* Journal of Natural Products, 2010. **73**(2): p. 263-266.

22. Prihanto, A.A. *Cytotoxic, antioxidant and antibacterial activity of methanol extract of Xylocarpusmoluccensis fruit husk*. in *The International Conference on Basic Science* 2011. Malang, Indonesia Galaxy Science publisher.

23. Wangensteen, H., et al., *Can Scientific Evidence Support Using Bangladeshi Traditional Medicinal Plants in the Treatment of Diarrhoea? A Review on Seven Plants.* Nutrients, 2013. **5**(5): p. 1757-1800.

24. Al-Abd, N.M., et al., *Recent Advances on the Use of Biochemical Extracts as Filaricidal Agents.* Evidence-based Complementary and Alternative Medicine : eCAM, 2013. **2013**: p. 986573.

25. Mahmud, I., et al., *Pharmacological and Ethnomedicinal Overview of Heritiera fomes: Future Prospects.* International Scholarly Research Notices, 2014. **2014**: p. 938543.

26. Chen, W., et al., *Absolute configurations of new limonoids from a Krishna mangrove, Xylocarpus granatum.* Fitoterapia, 2014. **94**: p. 108-13.

27. Choodej, S., et al., *Chamigrane Sesquiterpenes from a Basidiomycetous Endophytic Fungus XG8D Associated with Thai Mangrove Xylocarpus granatum.* Mar Drugs, 2016. **14**(7).

28. Gao, Q., et al., *Cipadesin A, a bioactive ingredient of Xylocarpus granatum, produces antidepressant-like effects in adult mice.* Neurosci Lett, 2016. **633**: p. 33-39.

29. Lakshmi, V., et al., *Gedunin and photogedunin of Xylocarpus granatum show significant anti-secretory effects and protect the gastric mucosa of peptic ulcer in rats.* Phytomedicine, 2010. **17**(8-9): p. 569-74.

30. Lakshmi, V., et al., *Antimalarial activity in Xylocarpus granatum (Koen).* Nat Prod Res, 2012. **26**(11): p. 1012-5.

31. Li, J., et al., *Andhraxylocarpins A-E: structurally intriguing limonoids from the true mangroves Xylocarpus granatum and Xylocarpus moluccensis.* Chemistry, 2012. **18**(45): p. 14342-51.

32. Li, M.Y., et al., *Granatumins A-G, limonoids from the seeds of a Krishna mangrove, Xylocarpus granatum.* J Nat Prod, 2009. **72**(12): p. 2110-4.

33. Misra, S., et al., *Gedunin and photogedunin of Xylocarpus granatum possess antifilarial activity against human lymphatic filarial parasite Brugia malayi in experimental rodent host.* Parasitol Res, 2011. **109**(5): p. 1351-60.

34. Pan, J.Y., et al., *Limonoids from the Seeds of a Hainan Mangrove, Xylocarpus granatum.* J Nat Prod, 2010. **73**(10): p. 1672-9.

35. Toume, K., et al., *Xylogranin B: a potent Wnt signal inhibitory limonoid from Xylocarpus granatum.* Org Lett, 2013. **15**(23): p. 6106-9.

36. Wu, Y.B., et al., *Xylomexicanins C and D, new mexicanolide-type limonoids from Xylocarpus granatum.* Biosci Biotechnol Biochem, 2013. **77**(4): p. 736-40.

37. Zhou, Z.F., et al., *Four phragmalin orthoesters from the Chinese mangrove Xylocarpus granatum.* Planta Med, 2014. **80**(11): p. 949-54.
